# Design and rationale of the Procalcitonin Antibiotic Consensus Trial (ProACT), a multicenter randomized trial of procalcitonin antibiotic guidance in lower respiratory tract infection

**DOI:** 10.1186/s12873-017-0138-1

**Published:** 2017-08-29

**Authors:** David T. Huang, Derek C. Angus, Chung-Chou H. Chang, Yohei Doi, Michael J. Fine, John A. Kellum, Octavia M. Peck-Palmer, Francis Pike, Lisa A. Weissfeld, Jonathan Yabes, Donald M. Yealy, on behalf of the ProACT Investigators, Michael Donnino, Peter Hou, Robert Sherwin, John Holst, Colleen Rafferty, Daniel Rodgers, William Dachman, Frank LoVecchio, Michael Filbin, Jonathan Fine, Jean Hammel, Matthew Exline, Lauren Southerland, Michael Kurz, David McCullum, Shahram Lotfipour, Gentry Wilkerson, Heather Prunty, Brian Suffoletto, Aaron Brown, Franziska Jovin

**Affiliations:** 1 The CRISMA (Clinical Research, Investigation, and Systems Modeling of Acute Illness) Center, Pittsburgh, PA USA; 2 Department of Critical Care Medicine, University of Pittsburgh, Room 606B Scaife Hall, 3550 Terrace Street, Pittsburgh, PA 15261 USA; 3 Department of Emergency Medicine, University of Pittsburgh, Pittsburgh, PA USA; 4 The MACRO (Multidisciplinary Acute Care Research Organization) Center, Pittsburgh, PA USA; 5 Division of General Internal Medicine, University of Pittsburgh, Pittsburgh, PA USA; 6 Division of Infectious Diseases, University of Pittsburgh, Pittsburgh, PA USA; 7 Center for Health Equity Research and Promotion, VA Pittsburgh Healthcare System, Pittsburgh, PA USA; 8 Department of Pathology, University of Pittsburgh, Pittsburgh, PA USA; 9 Eli Lilly and Company, Indianapolis, IN USA; 10 Statistics Collaborative, Inc., Washington, DC USA

**Keywords:** Procalcitonin, Biomarkers, Respiratory tract infections, Clinical trial, Anti-bacterial agents, Methods (MeSH)

## Abstract

**Background:**

Overuse of antibiotics is a major public health problem, contributing to growing antibiotic resistance. Procalcitonin has been reported to be commonly elevated in bacterial, but not viral infection. Multiple European trials found procalcitonin-guided care reduced antibiotic use in lower respiratory tract infection, with no apparent harm. However, applicability to US practice is limited due to trial design features impractical in the US, between-country differences, and residual safety concerns.

**Methods:**

The Procalcitonin Antibiotic Consensus Trial (ProACT) is a multicenter randomized trial to determine the impact of a procalcitonin antibiotic prescribing guideline, implemented with basic reproducible strategies, in US patients with lower respiratory tract infection.

**Discussion:**

We describe the trial methods using the Consolidated Standards of Reporting Trials (CONSORT) framework, and the rationale for key design decisions, including choice of eligibility criteria, choice of control arm, and approach to guideline implementation.

**Trial registration:**

ClinicalTrials.gov NCT02130986. Registered May 1, 2014.

## Background

Whether or not to administer antibiotics is a common and challenging clinical decision. Clinical presentations for infectious and non-infectious conditions overlap, and current diagnostic tests are inadequate. Given fears of untreated bacterial illness, clinicians often default to a decision to prescribe antibiotics. This pattern drives antibiotic overuse [1, 2] and resistance [3, 4], despite considerable efforts to change behavior [5–7]. Lower respiratory tract infection (LRTI) is arguably the most important example of this pattern. It is extremely common, but presentation is non-specific, making it difficult for clinicians to distinguish a bacterial from viral etiology or to distinguish LRTI from non-infectious conditions with similar signs and symptoms [8].

Host response to bacterial infection includes broad expression of procalcitonin from both immune and parenchymal cells, resulting in elevated serum concentration [9]. Viral infection does not appear to induce the same response [10]. The magnitude of elevation correlates with the severity of bacterial infection and decreasing concentrations over time correlate with resolution of infection [11–14]. Consequently, Mueller and colleagues tested whether the use of procalcitonin, folded into a treatment recommendation guideline, could help clinicians curb antibiotic use. Multiple European trials reported procalcitonin-guided care reduced antibiotic use in LRTI, with no apparent harm [15–18].

However, applicability to US practice is limited due to trial design features impractical in the US, between-country differences, and residual safety concerns [19]. For example, in the largest trial, the treating physicians enrolled patients in the emergency department (ED) and were only allowed to overrule procalcitonin guidance after consulting with the study center [15, 20]. In the US, ED volume and acuity are high and increasing [21, 22], and physicians highly value autonomy and resist protocolization [23]. Control group antibiotic duration and hospital length of stay were also twice that of current guideline recommendations [24] and US practice [25], and there is a growing trend towards short antibiotic courses [26]. In the only US trial, published in 2015, there was no significant difference in antibiotic use in a single center study of hospitalized LRTI patients randomized to standard care versus procalcitonin-guided care [27]. The incremental value of procalcitonin beyond best practice promotion of current guidelines [28, 29], and in clinically obvious cases [30], has therefore been questioned.

Current guidelines for procalcitonin guided LRTI care vary from low to moderately strong recommendation [31] to recommendation against routine adoption [32], reflecting indeterminate evidence. In February 2017, the US Food and Drug Administration approved procalcitonin to help determine if antibiotic treatment should be started or stopped in LRTI, based on a meta-analysis by the requesting sponsor (bioM*é*rieux, Marcy-l’Étoile, France), while noting the primary limitation of the meta-analysis was a lack of US clinical trial sites [33].

In November 2014, ProACT (Procalcitonin Antibiotic Consensus Trial, NCT02130986) began enrollment in the United States. ProACT seeks to determine the effect of a procalcitonin guideline on antibiotic exposure and adverse outcomes in clinically diagnosed LRTI, using a study design generalizable to US healthcare. This manuscript provides the trial methodology using the Consolidated Standards of Reporting Trials (CONSORT) framework [34–36], and discusses key design challenges and their resolution.

## Methods

### Trial methodology and rationale

ProACT is a patient-level, 1:1 randomized, parallel group, 14-center US trial comparing a procalcitonin-guided antibiotic prescribing guideline (implemented with basic reproducible strategies, including education, embedment into the electronic health record, and reminders) to usual care. We chose to test this guideline in a usual care environment where best practice exists and is promoted, in patient encounters with clinical uncertainty regarding antibiotic prescription, and with a design that embraces clinician autonomy. We summarize trial methods in Tables 1, 2, 3, 4 and 5. The following CONSORT-Methods sections provide additional details and context.

**Table 1:** Eligibility criteria

CONSORT	ProACT
Inclusion criteria	≥ 18 years of age A primary clinical diagnosis in the ED of acute LRTI (< 28 days duration) ^a^ Clinician willing to consider procalcitonin in antibiotic decision making
Exclusion criteria	Conditions where physicians are unlikely to withhold antibiotics Systemic antibiotics before ED presentation a. All prophylactic antibiotic regimens, or b. Received >1 dose within 72 h prior to ED presentation Current vasopressor use Mechanical ventilation (via endotracheal tube) Known severe immunosuppression ^b^ Accompanying non-respiratory infections Known lung abscess or empyema Conditions where PCT can be >0.25 μg/L without infection Chronic dialysis Metastatic cancer Surgery in the past 7 days (excluding minor surgery such as skin biopsy) Conditions rendering follow-up difficult Incarcerated or homeless Enrolled in ProACT in the past 30 days

*ED* emergency department, *LRTI* lower respiratory tract infection

^a^post-enrollment, LRTI is classified into the following categories (i) community acquired pneumonia, (ii) chronic obstructive pulmonary disease exacerbation, (iii) acute asthma exacerbation, (iv) acute bronchitis, (v) other LRTI

^b^known CD4 < 200/mm^3^, transplant patient on immunosuppressive medications, absolute neutrophil count <500 mm^3^

**Table 2:** Interventions

CONSORT	ProACT
Study arms	
Usual care	All care and decisions by existing care providers National LRTI guidelines disseminated No procalcitonin provided
Intervention	All care and decisions by existing care providers National LRTI guidelines disseminated Procalcitonin provided in ED, and if hospitalized, 6–24 h later, and on Days 3, 5, 7. ^a^
Standardization	Standardized teaching material at start-up and refresher meetings, frequently asked questions, access to coordinating center and principal investigator 24/7 Study website, center visits and newsletters Center monitoring
Adherence	Regular adherence reports of procalcitonin sample time collection, time to clinician notification, procalcitonin guideline adherence, and feedback to individual centers

*ED* emergency department, *LRTI* lower respiratory tract infection

^a^serial blood draws only occur in hospitalized patients on antibiotics

**Table 3:** Outcomes

CONSORT	ProACT
Outcomes	
Primary	Total antibiotic exposure, defined as the total number of antibiotic-days by Day 30 ^a^ A combined endpoint of adverse outcomes that could be attributable to withholding antibiotics in LRTI, that occur by study Day 30 ^b^: i. death ii. septic shock (vasopressor use for >1 h) iii. Mechanical ventilation (via endotracheal tube) iv. renal failure (KDIGO, stage 3) [50] v. lung abscess/empyema vi. development of pneumonia in non-pneumonia LRTI vii. Subsequent hospitalization
Secondary	Antibiotic initiation by the initial ED clinician Hospital length of stay 90d and 1 year mortality Intensive care unit admission Subsequent ED visits Quality of life (Airway Questionnaire 20) [57]
Data quality methods	Standardized data collection and recording Web-based DCF with built-in logic checks, automatic data queries, and streamlined user interface Periodic DCF checks to monitor data irregularities and protocol compliance Study coordinator DCF training and periodic conference calls Center monitoring visits and review of source documents

*LRTI* lower respiratory tract infection, *KDIGO* Kidney Disease Improving Global Outcomes, *ED* emergency department, *DCF* data collection form

^a^We define an antibiotic-day as each day a participant receives any oral or intravenous antibiotics, excluding antibiotics given for non-infectious indications (e.g. rifaximin for hepatic encephalopathy) and antivirals

^b^primary safety outcome

**Table 4:** Sample size determination and interim analyses

CONSORT	ProACT
Sample size	1664
Determination	H1o: Procalcitonin guideline implementation does not reduce antibiotic exposure by Day 30. (superiority) H2o: Procalcitonin guideline implementation increases the proportion of subjects who experience a composite endpoint of adverse outcomes by Day 30, by ≥4.5%. (non-inferiority) ▪ Sample size is driven by H2o ▪ 4.5% non-inferiority margin ▪ ≥ 80% power, 1-sided alpha of 0.05 ▪ Lost to follow up and composite endpoint rates at 2nd interim data safety monitoring board meeting at ~2/3 accrual (April 2017)
Interim analyses and stopping rules	Two interim analyses and one final analysis, approximately evenly spaced O-Brien and Fleming stopping boundaries

**Table 5:** Randomization, blinding, and statistical methods

CONSORT	ProACT
Randomization	
Sequence generation	Patient-level, permuted block design Stratified by center, race, age Randomized equally to each study arm
Allocation concealment	Central Web-based randomization, accessible 24 h/day
Implementation	Local center staff enroll patients via Web-based randomization system Web-based system then assigns patients to trial arm, based on computer generated allocation sequence
Blinding	Statistical analysis and post-discharge outcome assessment staff are blinded to study arm By arm outcome data restricted to unblinded statistician and data safety monitoring board
Statistical methods	Intention-to-treat, as per pre-established analysis plan (primary analysis) Per-protocol analysis, where procalcitonin guideline is followed

### Participants (patients)

#### Inclusion criteria

Study staff enroll adult ED patients with a primary clinical diagnosis of acute LRTI, where the treating clinician is willing to consider procalcitonin in antibiotic decision making (Table 1). By targeting encounters where the clinician has not already decided to give or withhold antibiotics, we seek to enroll LRTI cases where clinical uncertainty exists.

#### Exclusion criteria

We exclude patients with conditions where (1) physicians are unlikely to withhold antibiotics (e.g., patients receiving endotracheal ventilation), (2) procalcitonin can be elevated without bacterial infection (e.g., recent surgery), and (3) follow-up would be difficult (e.g., prisoners, homeless) (Table 1).

### Participants (centers)

We chose centers with evidence of commitment to LRTI quality care. All centers had achieved >96% compliance with all Joint Commission pneumonia core measures. We chose centers and center principal investigators based on clinical research experience, clinical expertise in LRTI management, ED volume, projected recruitment, ability to execute study procedures both in ED and in hospital, absence of routine procalcitonin use, and geographic diversity (Appendix 1). Each center has clinical, laboratory, and health records systems that allow prompt notification of procalcitonin results. Centers are mostly urban academic hospitals.

### Interventions

#### Study arms

In both arms (Table 2) the bedside clinicians retain complete autonomy for all patient care decisions, and we disseminate national LRTI guidelines.

**Fig. 1:**
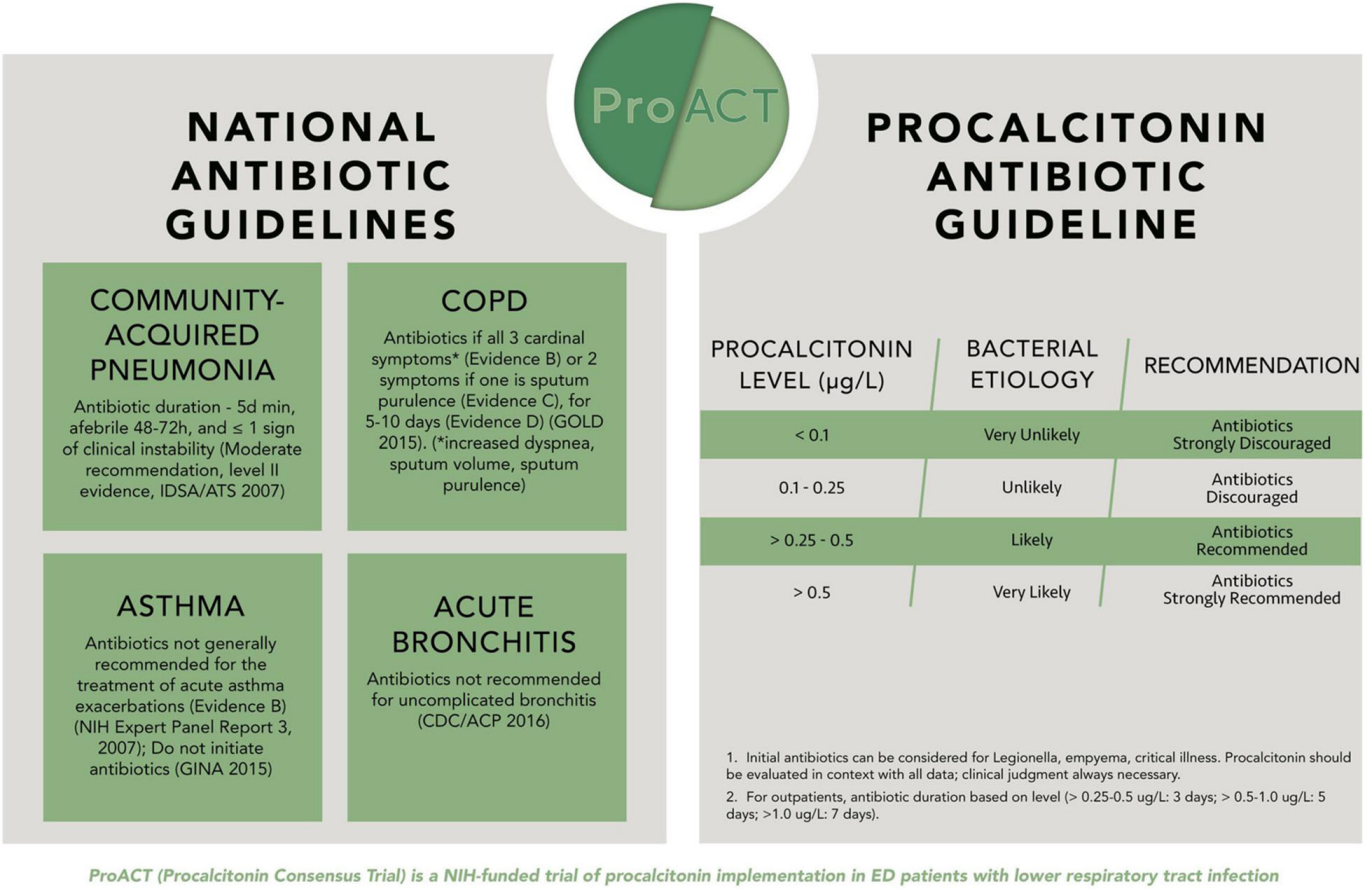
ProACT guidelines. The ProACT Coordinating Center provided posters of this Figure to all centers. Other study education, in-service training, and promotion materials contain the same content

We incorporate LRTI guideline recommendations in all study lectures, posters, and promotion tools. We provide relevant excerpts from the following guidelines: chronic obstructive pulmonary disease – Global Initiative for Chronic Obstructive Lung Disease [37]; asthma – National Asthma Education and Prevention Program’s Expert Panel Report 3 [38], Global Initiative for Asthma [39]; acute bronchitis – Center for Disease Control/American College of Physicians guidelines [40]; community-acquired pneumonia – Infectious Diseases Society of America/American Thoracic Society guidelines [24] (Fig. 1, left panel).

#### Intervention

The intervention consists of reporting the procalcitonin results and guideline (Fig. 1, right panel) to clinicians. The same procalcitonin guideline is provided with both the initial and serial procalcitonin measurements - withhold or cease antibiotics if low, administer or continue if high. Participants have blood drawn for a procalcitonin level in the ED, and if hospitalized, 6–24 h after the initial ED blood draw, and on Days 3, 5, and 7 if still in hospital and on antibiotics.

We used several implementation strategies, centered around a primary message of “Please look at the procalcitonin value and guideline recommendation, but the final antibiotic decision is entirely yours.” With coordinating center support and tools, each site conducted background education and in-service training prior to study launch, and during the trial. All clinicians involved in antibiotic prescription for LRTI, including residents, hospitalists, primary care physicians, nurse practitioners, and physician assistants were targeted for in-service training. To promote easy reminders we embedded the procalcitonin information into the electronic health record of each site where feasible (Appendix 2). This approach mimics how clinicians often receive laboratory test data with range-based interpretation, such as with troponin and d-dimer. Lastly, coordinators are trained to identify the key clinician with primary responsibility for antibiotic decision making, inform the clinician the procalcitonin information is available, and not otherwise influence care. In the ED, coordinators ensure clinicians quickly (< 1 h goal) receive the procalcitonin information. For patients admitted to hospital, coordinators inform hospital clinicians of the ED procalcitonin information and when serial procalcitonin results are available. Our intent is to mimic how a hospital might typically deploy quality improvement staff when introducing a new intervention. The final decision to order antibiotics is at the discretion of the treating clinician.

#### Usual care

Study personnel solely collect data and biologic specimens in usual care arm participants. We also sought to minimize contamination (procalcitonin use in usual care). At study launch, no center routinely used procalcitonin, only 2 centers had procalcitonin clinically available, and no LRTI guidelines recommended routine clinical use of procalcitonin.

#### Standardization

To standardize study procedures, we provide standardized training and materials plus continuous coordinating center support. We conducted a group investigator and coordinator training meeting at study launch, and individual sessions for two centers that were added subsequently. Training materials are available on the study website. Regular center visits, newsletters, around-the-clock coordinating center access, center monitoring, protocol delivery and procalcitonin guideline adherence reports and feedback were used to further enhance standardization.

To standardize procalcitonin measurement, bioMérieux provided the procalcitonin assay equipment, installation, and in-service training. We provided centers with a packet that outlined test result reporting procedures, troubleshooting procedures, frequently asked questions, and study contact information. Each center’s Clinical Laboratory Improvement Amendments (CLIA) certified laboratory measured procalcitonin from plasma [41] or serum [42] samples, using a 1-step enzyme immunoassay sandwich method on bioMérieux VIDAS or mini-VIDAS immunoanalyzers with an analytic range of 0.05–200 ng/ml [43]. To ensure accurate testing, each center performed standard instrument calibration procedures, analyzed two levels of quality control materials with each sample run, and at minimum biannually assessed assay linearity [44, 45]. Additionally, twelve centers annually participated in a College of American Pathologists (CAP) proficiency testing program for procalcitonin, the American Proficiency Institute program, or conducted external peer (inter-laboratory) testing [44, 46]. Procalcitonin levels remain stable under multiple freeze/thaw, storage, and temperature conditions [41, 43, 47].

#### Adherence

Our overall adherence approach is similar to many quality improvement programs. This design balances enforcement strategies not reproducible in routine care with a completely hands-off strategy that risks trial failure due to study unawareness [48, 49].

For each study procalcitonin blood draw, we track the times for sample collection, and times from sample collection to clinician notification of procalcitonin information. We promote adherence to the study intervention with regular feedback to each center, and identify solutions for rectifying non-adherence.

We track clinician adherence to the procalcitonin antibiotic guideline. If antibiotics are prescribed or continued when procalcitonin is low, coordinators query the clinician and record the reasons for non-adherence. We promote adherence to the procalcitonin guideline with regular feedback and discussion with each center. To increase study awareness and guideline adherence, centers in-service trained all clinician groups with primary responsibility for antibiotic prescription for LRTI. Upon discharge from ED or hospital, participants receive a packet that includes a letter to their primary care physician with a study synopsis, their last procalcitonin result, and the procalcitonin guideline.

### Outcomes

#### Primary

Our primary outcome is total antibiotic exposure, defined as the total number of antibiotic days by Day 30 (Table 3). We define an antibiotic day as each day a subject receives any oral or intravenous antibiotic, excluding antibiotics given for non-infectious indications (e.g. rifaximin for hepatic encephalopathy) and antiviral agents.

Our primary safety outcome is a combined endpoint of adverse outcomes that could be attributable to withholding antibiotics in LRTI. The individual outcomes are death, septic shock (vasopressor use), mechanical ventilation via endotracheal tube, renal failure (Kidney Disease: Improving Global Outcomes stage 3 – new renal replacement therapy, tripling of baseline creatinine, or serum creatinine ≥4.0 mg/dL [50]), lung abscess/empyema, development of pneumonia in non-pneumonia LRTI, and hospital readmission, by day 30. Occurrence of one or more of these outcomes by day 30 will count as reaching the primary safety outcome. We will also examine each outcome individually. Although these outcomes are of different gravity, if any one of them were to occur, clinicians and patients would likely believe the procalcitonin guideline failed, and that antibiotics should have been provided. We therefore use a combined endpoint to capture each such adverse outcome.

#### Secondary

Secondary outcomes include antibiotic initiation by the initial ED clinician, hospital length of stay, 90 day and 1 year mortality, ICU admission and subsequent ED visits by Day 30, and quality of life at day 15 and day 30 (Table 3).

#### Data quality methods

We monitor data quality using web-based data collection, automated queries, and center monitoring visits, and provide structured data collection training to centers prior to study initiation. Coordinating center staff blinded to study arm conduct follow-up calls to determine post-discharge outcomes, using a structured interview process. To facilitate participant recall and retention, we conduct calls at both day 15 and day 30, provide an antibiotic diary at discharge, and obtain multiple contact phone numbers at enrollment. In 2016, we added text messaging, email, and postal mail follow-up methods.

### Sample size

#### Determination

ProACT will test the following two null hypotheses.

- *H1o*: Procalcitonin guideline implementation does not reduce or increase antibiotic exposure by Day 30.
- *H2o*: Procalcitonin guideline implementation increases the proportion of subjects who experience a composite endpoint of adverse outcomes by Day 30, by ≥4.5%.

We computed our original sample size of 1514 participants based on the difference in proportions of the composite adverse outcomes endpoint between the two arms in H2o. Our power calculations accounted for two interim analyses at approximately 1/3 and 2/3 enrollment with stopping boundaries calculated using the O’Brien and Fleming method, ≥ 80% power to reject H2o, significance at the 0.05 level, a predefined 4.5% non-inferiority margin, and assuming an 11% adverse outcomes by Day 30 event rate in the usual care arm [51, 52], and ~10% loss to follow up rate. We thus calculated sample size under assumptions of event and lost to follow up rates, and also prospectively monitored both rates with an intent to recalculate and adjust sample size as necessary. In April 2017, the data and safety monitoring board held the second interim analysis meeting, and approved an increase of sample size to 1664 participants (Table 4).

The CONSORT extension for noninferiority trials notes that an overly large non-inferiority margin risks accepting a truly inferior treatment as noninferior, while a very small margin may produce inconclusive results, requiring an extremely large trial to achieve adequate power [36]. We chose the smallest non-inferiority margin feasible within our funding structure, approximately half that of the margins recommended by the Infectious Diseases Society of America recommendation for non-inferiority trials assessing antibiotic treatment for community-acquired pneumonia, and the margins used in two large trials of procalcitonin antibiotic guidance [15, 53].

#### Interim analyses and stopping rules

We submit data to an independent, multidisciplinary data safety and monitoring board for interim analyses on a predefined schedule and with a priori stopping rules. Before trial completion, only the board and designated study statistician see per-arm outcome data; the board may recommend stopping enrollment for any reason including efficacy, harm, or futility.

Pre-established statistical plans and oversight committee charters mitigate concerns of spurious early cessation [54].

### Randomization

#### Sequence generation

ProACT randomizes at the patient level, with 1:1 study arm allocation using a computer generated, permuted block design, stratified by center, race, and age (Table 5).

#### Allocation concealment

We assure concealment via an automated centralized assignment system. Only after enrollment does the system assign a study arm.

#### Implementation

All participants who give consent for participation, fulfill inclusion criteria, and have no exclusion criteria are randomized. Coordinators enter participant information into the web-based data collection form and receive from the randomization system a study ID number and treatment assignment. There is no influence on randomization by the principal investigator, center study team, or the ProACT coordinating center.

### Blinding

Due to the nature of the intervention, neither the treating clinician nor the study staff can be blinded to allocation. Statistical analysis and post-discharge outcome assessment staff are blinded to allocation. We restrict access to unblinded data to a designated study statistician and data safety and monitoring board.

### Statistical methods

An independent statistician blinded to treatment allocation will conduct analyses using a pre-established statistical plan. The primary analysis is an intent-to-treat (ITT) analysis, for both hypotheses. ITT can bias towards no difference, which may lead to a false rejection of H2o, which uses a non-inferiority design. We therefore will also perform per-protocol analyses where the procalcitonin guideline was followed, as per CONSORT recommendations [36]. We will summarize baseline characteristics by study arm, and will test the primary hypotheses for significance at the 0.05 level.

For H1o, we will compare the mean number of total antibiotic-days by Day 30 using two-sample t-test, or a nonparametric counterpart if data distribution is not normal, and report two-sided *p*-values for significance. In the U.S., antibiotic use for LRTI is high, and our design excludes common conditions where procalcitonin can be high without infection. Given these two conditions, we believed procalcitonin would not increase antibiotic use over an already high baseline, and initially chose one-sided significance testing. To be conservative and allow for the possibility of increased antibiotic use in the intervention arm, we will conduct and report two-sided *p*-values. As sample size is driven by the larger requirements of noninferiority testing for H2o, we are well powered to test H1o.

For H2o, we will compare the difference in proportions of the composite adverse outcomes endpoint, relative to a 4.5% non-inferiority margin, and construct a two-sided 95% confidence interval for the difference in proportions. We will declare non-inferiority if the upper limit of the confidence interval is below 4.5%. Non-inferiority hypothesis testing is one-sided. We will report results in accordance with the CONSORT statements.

## Discussion

ProACT is the first multicenter U.S. randomized trial of procalcitonin. In designing the trial, we considered three key issues – choice of eligibility criteria, choice of control arm, and approach to guideline implementation.

Tests should only be obtained if results may change management [55]. We therefore designed eligibility criteria to select patients whose care could reasonably be impacted by procalcitonin guidance. In particular, we targeted those patients for whom clinicians were willing to consider procalcitonin in their antibiotic decision making. In other words, patient encounters where a degree of clinical indecision exists, and thus an additional diagnostic might assist decision making, rather than only add cost.

Trials should test novel interventions on a background of “best care”. We chose centers with evidence of commitment to LRTI quality care, and disseminated national LRTI guidelines to promote best practice. This approach balances the control arm extremes of “wild type” usual care, versus an “active control” arm with interventional enforcement, consistent with the NIH conference on Considering Usual Medical Care in Clinical Trial Design recommendations [56].

We chose a guideline implementation approach generalizable to U.S. clinical practice. A key difference between ProACT and the largest European LRTI trial is that the guideline recommendation is not deployed using coordinating center “enforcement methods” [15, 20]. Instead, to facilitate implementation into routine care, we provide background education and in-service training, embed the procalcitonin results and guideline into the electronic health records and clinical laboratories of study centers, and use coordinator reminders to ensure information receipt. This approach more closely reflects how procalcitonin guidance would be received and used by clinicians in U.S. practice.

## Conclusion

ProACT will provide generalizable evidence on the impact of a procalcitonin guideline, implemented with basic reproducible strategies, on antibiotic exposure and safety in U.S. patients with lower respiratory tract infection.

## Appendix 1

**Table 6:** ProACT Centers and Investigators

Center	# hospital beds	Urbanicity	Teaching status	Ownership	City, State
Beth Israel Deaconess Medical Center	602	Urban	Large teaching	Nonprofit	Boston, MA
Brigham and Women’s Hospital	763	Urban	Large teaching	Nonprofit	Boston, MA
Detroit Receiving Hospital	225	Urban	Large teaching	Profit	Detroit, MI
Essentia Health St. Mary’s Medical Center	545	Rural	Small teaching	Nonprofit	Duluth, MN
Hershey Medical Center	454	Urban	Large teaching	Nonprofit	Hershey, PA
Maricopa Medical Center	275	Urban	Large teaching	Government	Maricopa. AZ
Massachusetts General Hospital	941	Urban	Large teaching	Nonprofit	Boston, MA
Norwalk Hospital	261	Urban	Large teaching	Nonprofit	Norwalk, CT
Ohio State University Hospital	850	Urban	Large teaching	Government	Columbus, OH
University of Alabama Hospital	997	Urban	Large teaching	Government	Birmingham, AL
University of California Irvine Medical Center	350	Urban	Large teaching	Government	Irvine, CA
University of Maryland Medical Center	771	Urban	Large teaching	Nonprofit	Baltimore, MD
UPMC Mercy	419	Urban	Large teaching	Nonprofit	Pittsburgh, PA
UPMC Presbyterian	795	Urban	Large teaching	Nonprofit	Pittsburgh, PA

We defined teaching status using the resident-to-bed ratio, classifying hospitals as nonteaching if they had no resident trainees, small teaching if the ratio was more than zero and less than 0.2, and large teaching if the ratio was 0.2 or greater [58]

Beth Israel Deaconess Medical Center: Michael Donnino; Brigham and Women’s Hospital: Peter Hou; Detroit Receiving Hospital: Robert Sherwin; Essentia Health St. Mary’s Medical Center: John Holst; Hershey Medical Center: Colleen Rafferty, Daniel Rodgers; Maricopa Medical Center: William Dachman, Frank LoVecchio; Massachusetts General Hospital: Michael Filbin; Norwalk Hospital: Jonathan Fine, Jean Hammel; Ohio State University Hospital: Matthew Exline, Lauren Southerland; University of Alabama Hospital: Michael Kurz, David McCullum; University of California Irvine Medical Center: Shahram Lotfipour; University of Maryland Medical Center: Gentry Wilkerson; University of Pittsburgh Medical Center Mercy Hospital: Heather Prunty, Brian Suffoletto; University of Pittsburgh Medical Center Presbyterian Hospital: Aaron Brown, Franziska Jovin

## Appendix 2

**Table 7:** Procalcitonin information delivery methods

Center	PCT Delivery Method	EHR Type	Laboratory Information System
Beth Israel Deaconess Medical Center	Paper	N/A	N/A
Brigham and Women’s Hospital	Paper	N/A	N/A
Detroit Receiving Hospital	Paper	N/A	N/A
Essentia Health St. Mary’s Medical Center	Electronic Health Record	Epic	Soft Lab
Hershey Medical Center	Electronic Health Record	Cerner	Sunquest
Maricopa Medical Center	Electronic Health Record	Epic	Epic Beaker
Massachusetts General Hospital	Electronic Health Record	Epic	Sunquest
Norwalk Hospital	Electronic Health Record	Epic	Sunquest
Ohio State University Hospital	Electronic Health Record	Epic	Sunquest
University of Alabama Hospital	Electronic Health Record	IMPACT	IMPACT
University of California Irvine Medical Center	Paper	N/A	N/A
University of Maryland Medical Center	Paper	N/A	N/A
UPMC Mercy	Electronic Health Record	Cerner	Sunquest
UPMC Presbyterian	Electronic Health Record	Cerner	Sunquest

*PCT* procalcitonin, *EHR* electronic health record

## Abbreviations

CAP: College of American Pathologists
CLIA: Clinical Laboratory improvement amendments
CONSORT: Consolidated standards of reporting trials
ED: Emergency department
ITT: intent-to-treat
LRTI: Lower respiratory tract infection
ProACT: Procalcitonin antibiotic consensus trial
